# Supplementary figures and images for: Host-Encoded Aminotransferase Import into the Endosymbiotic Bacteria Nardonella of Red Palm Weevil
Source: Insects. 2024 Jan 5;15(1):35. doi: 10.3390/insects15010035 (PMC10816905; doi:10.3390/insects15010035)

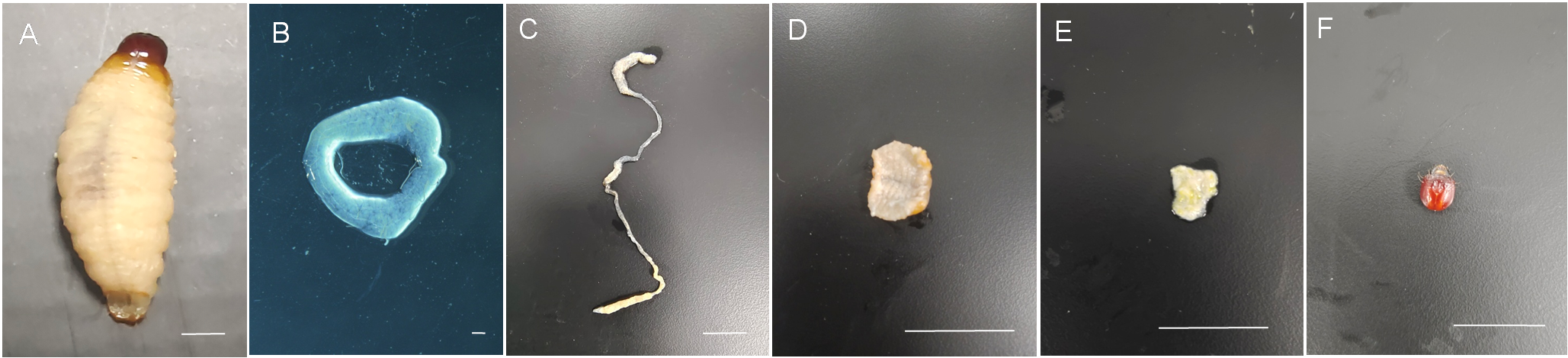

Supplement: Supplementary file 1 [file insects-15-00035-s001.zip › insects-2781344-Figure S1 supplementary.tif]
